# A novel method using body surface steel ball as a reference scale to measure the left atrial appendage for optimal selection of Watchman device

**DOI:** 10.3389/fcvm.2025.1464567

**Published:** 2025-03-14

**Authors:** He Jin, Mingjun Feng, Xianfeng Du, Binhao Wang, Yibo Yu, Guohua Fu, Caijie Shen, Huimin Chu

**Affiliations:** Cardiac Arrhythmia Center, The First Affiliated Hospital of Ningbo University, Ningbo, China

**Keywords:** left atrial appendage closure, Watchman, steel ball, sheath, TEE

## Abstract

**Background:**

Left atrial appendage closure (LAAC) is an alternative to oral anticoagulation for stroke prevention in non-valvular atrial fibrillation (AF). Selecting the appropriate size of Watchman device is very important intra-procedure. There are several methods have been reported to measure the left atrial appendage (LAA), but each of them has its limitations.

**Objective:**

We investigated the efficacy and safety of using the novel “steel ball method” compared to using the traditional “sheath method” and TEE during procedure of LAAC with Watchman device in AF patients.

**Methods:**

Patients with atrial fibrillation who underwent LAAC with Watchman device at The First Affiliated Hospital of Ningbo University from January 2018 to December 2021 were retrospectively analyzed. A 10 mm-diameter steel ball was placed on patient's body surface at the pulmonary valve auscultation zone before procedure. The maximum LAA ostium diameter, maximum LAA depth, and 1st sheath marker band length were measured under x-ray fluoroscopy, using the delivery sheath, pigtail sheath, and steel ball as references, respectively, which we called the delivery sheath group, pigtail sheath group and steel ball group. The maximum LAA ostium diameter and maximum LAA depth were also measured by TEE. All Watchman devices were selected based on the measurement of LAA by “steel ball method”. The position of Watchman device and presence of peri-device leakage (PDL) were assessed using transesophageal echocardiography (TEE) before and after the release.

**Results:**

Eventually a total of 169 patients [63.3% male, age 69 (44–87) years, 73.4% persistent or permanent AF, CHA2DS2-VASc score 4.31 ± 1.54, HAS-BLED score 2.74 ± 1.15, left atrial diameter 44.09 ± 7.55 mm] underwent Watchman device implantation successfully were enrolled. The mean maximum LAA ostium diameter measured in steel ball group (24.73 ± 3.39 mm) was significantly higher than that in delivery sheath group (20.04 ± 3.24 mm, *p* < 0.001) and pigtail sheath group (22.48 ± 3.74 mm, *p* < 0.001), while was not significantly different from the results measured by TEE (24.39 ± 4.13 mm, *p* = 0.176). The difference between 1st sheath marker band length measured in steel ball group and the true length (21 mm) was 0.29 ± 0.61 mm, which was significantly less than that in delivery sheath group (4.22 ± 1.42 mm) and pigtail sheath group (2.17 ± 1.90 mm) (both *p* < 0.001). Finally, the success rate of Watchman device implantation is 98.8%, with no serious intra-procedure complication. 2 patients (1.2%) occurred pericardial tamponade after procedure. 98.8% and 97.0% of patients had either no or slight (≤3 mm) PDL with immediate and 45 days post-procedural TEE scans, respectively. Device-related thrombosis (DRT) was detected in 1 patient (0.6%) and 2 patients (1.2%) had ischemic stroke during follow-up.

**Conclusions:**

In LAAC, the novel method using body surface steel ball as a reference scale to measure the left atrial appendage and guide the selection of Watchman device is accurate, effective, and safe. The size of Watchman device may be too small if selection is based on the measurement results with “sheath method”, which can lead to unsatisfactory outcome of the procedure.

## 1 Introduction

Ischemic stroke is a serious complication in patients with atrial fibrillation (AF), and the risk is 4–5 times higher than that in non-AF patients, resulting in nearly 20% of deaths and 60% of disabilities (1). Thrombus is most likely to occur in the left atrial appendage (LAA) with AF patients, and left atrial appendage closure (LAAC) is an alternative to oral anticoagulation for stroke prevention in non-valvular AF (2, 3). Selecting the appropriate size of Watchman device is very important because undersizing the device may lead to Device-related thrombosis (DRT) and peri-device leakage (PDL), while oversizing may lead to LAA perforation and cardiac tamponade.

To determine the size of Watchman device to be implanted, it is necessary to measure the relevant size of the LAA, such as the maximum LAA ostium diameter and maximum LAA depth. As we know, the traditional “sheath method” of measuring the LAA size during procedure is based on the diameter of the delivery sheath or pigtail sheath shown on x-ray as a reference scale in some electrophysiology centers in China. The measured size of the LAA using “sheath method” may be affected by several factors (e.g., material characteristics of sheath, sheath deformability, x-ray projection angle, and compressibility in the body cavity, etc.), which may lead to inaccurate measurement results (4). There are some other methods such as using pre-procedural left atrial computed tomography angiography (CTA), intra-procedural transesophageal echocardiography (TEE) under general anesthesia, or intra-procedural ICE to measure the size of LAA (5–8). However, each of these methods had its limitations in terms of measurement accuracy, patient physiological state tolerance, ease of surgical operation, popularity of the technology, and economic perspective.

The novel “steel ball method” uses the diameter of the body surface steel ball as a reference scale, which may have a potential advantage because the steel ball is a non-deformable, non-compressed, x-ray impermeable sphere and its diameter shown on x-ray is the same in any x-ray projection angle. We investigated the efficacy and safety of using the novel “steel ball method” compared to the traditional “sheath method” and TEE during procedure of LAAC with Watchman device in AF patients.

## 2 Methods

A total of 171 patients with atrial fibrillation who underwent LAAC with Watchman device (Boston Scientific, USA) at The First Affiliated Hospital of Ningbo University from January 2018 to December 2021 were retrospectively analyzed, and 169 of them were successfully implanted with WATCHMAN. The vast majority of patients enrolled were those who had contraindications or were unwilling to accept long-term oral anticoagulation. Pre-procedural TEE or CTA was performed in all patients to exclude atrial thrombosis. We also collected standard demographic, clinical characteristics, and baseline transthoracic echocardiographic parameters.

### 2.1 Pre-procedural preparation and intra-procedural measurements

**Figure 1:**
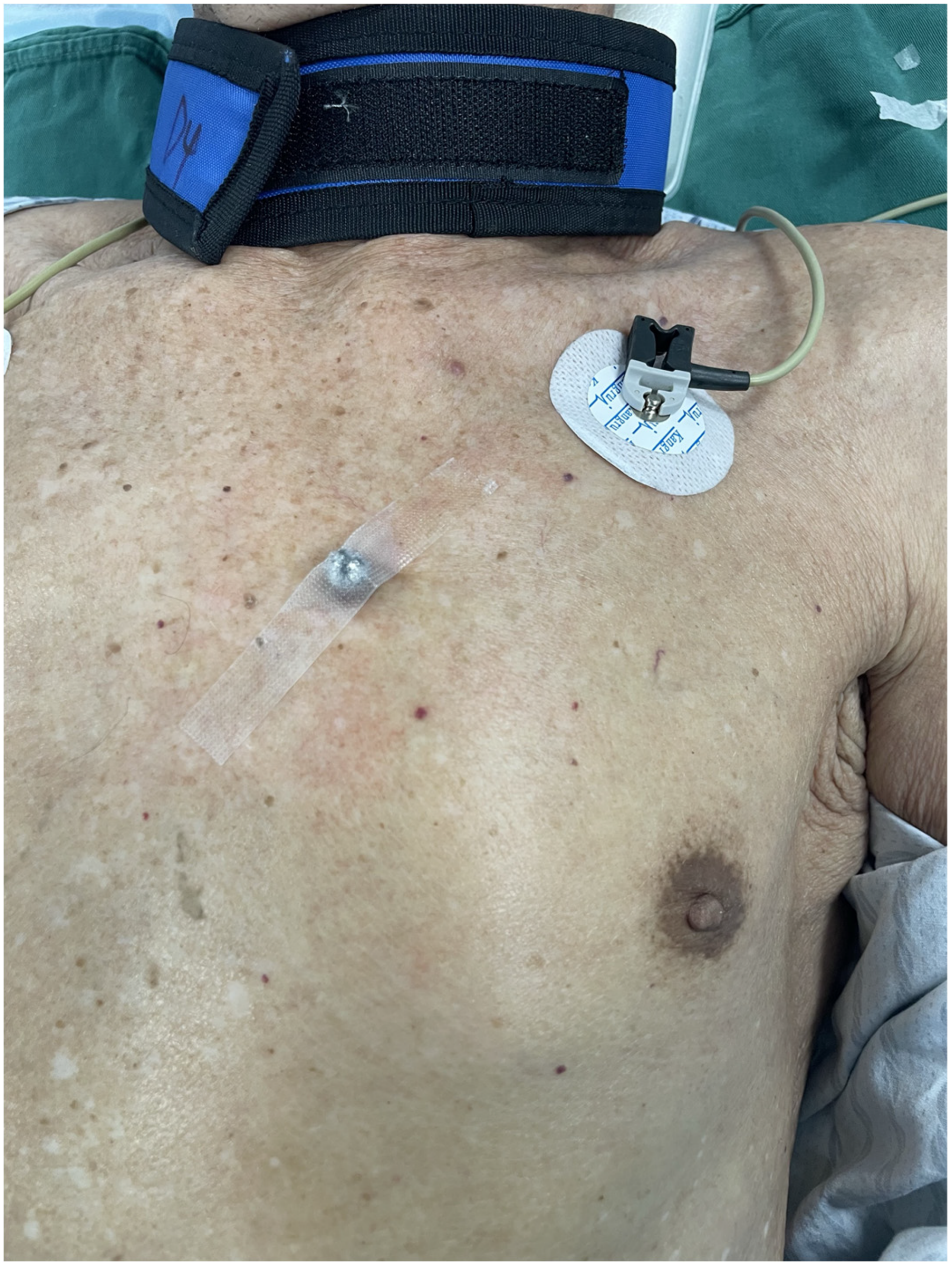
A 10 mm-diameter steel ball was placed on patient's body surface at the pulmonary valve auscultation zone (the second intercostal space at the left edge of the sternum) before procedure.

**Figure 2:**
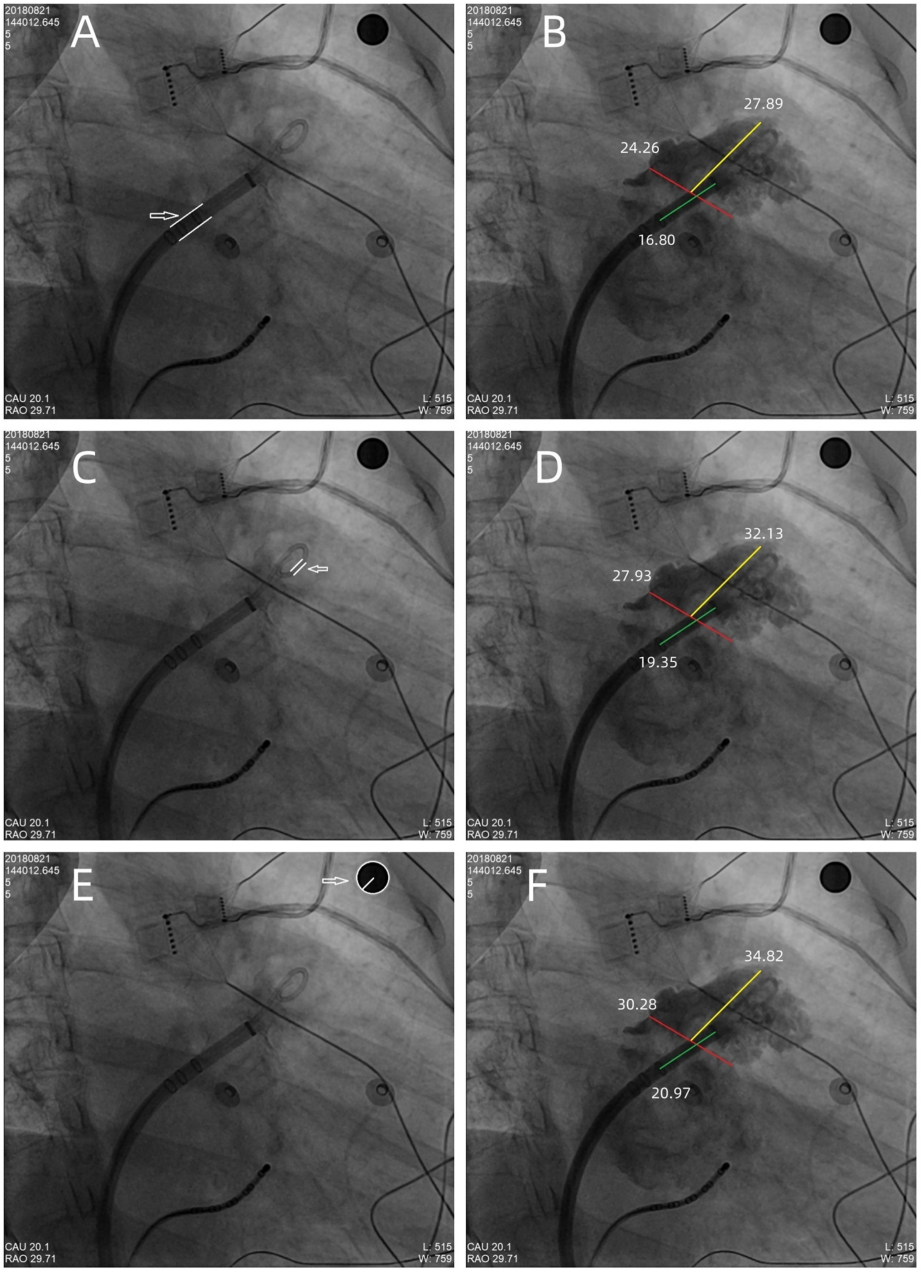
The maximum LAA ostium diameter, maximum LAA depth, and 1st sheath marker band length were measured under x-ray fluoroscopy, using the delivery sheath, pigtail sheath, and steel ball as references, respectively. **(A,B)** The double white lines pointed by the white arrow showed the 12Fr inner diameter delivery sheath (4 mm in diameter) as reference scale, the red line showed the measurement of maximum LAA ostium diameter (24.26 mm), the yellow line showed the measurement of maximum LAA depth (27.89 mm), and the green line showed the measurement of 1st sheath marker band length (16.80 mm). Similarly, in **(C,D)**, the double white lines showed the 5Fr delivery sheath (1.67 mm in diameter) as reference scale, the red line, yellow line, and the green line showed the measurement of maximum LAA ostium diameter (27.93 mm), maximum LAA depth (32.13 mm), and 1st sheath marker band length (19.35 mm), respectively. **(E,F)** The white arrow showed the steel ball (10 mm in diameter) as reference scale, the red line, yellow line, and green line showed the measurement of maximum LAA ostium diameter (30.28 mm), maximum LAA depth (34.82 mm), and 1st sheath marker band length (20.97 mm), respectively. In this case, a 33 mm Watchman device was successfully implanted with satisfactory results.

**Figure 3:**
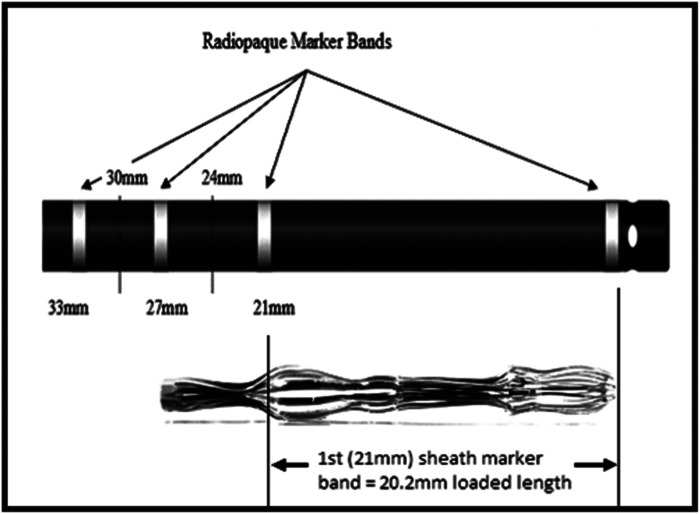
The 1st sheath marker band length on delivery sheath of Watchman device.

A 10-mm-diameter steel ball was placed on the patient's body surface at the pulmonary valve auscultation zone (the second intercostal space at the left edge of the sternum) before procedure in all 169 enrolled patients (Figure 1). Trans-septal puncture was guided by x-ray fluoroscopy, following femoral venous access under local anesthesia. Intravenous heparin was administered at a dose of 100 IU/kg to maintain the activated clotting time (ACT) >250 s intra-procedural. The 12Fr inner diameter delivery sheath and 5Fr pigtail sheath were used during all the procedures, and the size and shape of LAA were clearly visualized by injecting iodinated contrast medium under x-ray fluoroscopy. The maximum LAA ostium diameter, maximum LAA depth and 1st sheath marker band length were measured with calibrate measurement software at several different x-ray fluoroscopic angles if necessary (including at RAO30° + CAU20°, CAU20°, RAO45°, etc., usually at RAO30° + CAU20°), using the delivery sheath, pigtail sheath, and steel ball as references, respectively (Figure 2). The maximum LAA ostium diameter and maximum LAA depth were also measured by TEE. The 1st sheath marker band on delivery sheath of WATCHMAN device is length-fixed (21 mm) and impervious to x-ray (9) (Figure 3). The smaller the difference between the measured 1st sheath marker band length and 21 mm, the more accurate the measurement considered to be.

### 2.2 Watchman device implantation and post-procedure

All Watchman devices were selected based on the measurement of LAA by “steel ball method” and released after satisfying the “PASS” principle (10). All implantations of Watchman device were performed by experienced operators. The position of Watchman device and the presence of PDL were assessed using TEE by experienced ultrasound physicians before and after the release in each case. Meanwhile, tug testing was performed in all patients under x-ray fluoroscopy or TEE to ensure device stability before final release. All patients underwent transthoracic echocardiography the day after the procedure to rule out complications such as pericardial effusion. The routine post-procedural anticoagulation strategy was taking Warfarin or non-vitamin K antagonist oral anticoagulant (Rivaroxaban/Dabigatran) for 45 days, and TEE was performed again at 45 days post-procedure. If there was no DRT on Watchman device as well as the PDL ≤ 5 mm, anticoagulants were discontinued and replaced with dual antiplatelets until 6 months post-procedure, after that mono-antiplatelet therapy would be given. Otherwise, the duration of anticoagulation would be prolonged appropriately according to the experience of electrophysiologists.

### 2.3 Statistical analysis

The Kolmogorov–Smirnov test was used to assess for normal distribution. Continuous variables were expressed as mean ± standard deviation, or as median and ranges. Categorical variables were reported as frequencies and percentages. Comparisons between groups were made using the Chi-square or Fisher exact test for categorical variables and the matched samples *t*-test or Wilcoxon test for continuous data. A two-sided *P* value < 0.05 was considered statistically significant. Statistical analyses were performed using SPSS software (version 26.0, IBM).

## 3 Results

Eventually, 169 patients who underwent Watchman device implantation successfully were included in the study. The baseline demographics and clinical characteristics of the study cohort are shown in Table 1. 63.3% of patients were male, the median patient age was 69 (44–87) years, persistent or permanent AF in 73.4% of patients, the mean CHA2DS2-VASc score was 4.31 ± 1.54, and the mean HAS-BLED score was 2.74 ± 1.15. The mean left atrial diameter was 44.09 ± 7.55 mm, and the mean left ventricular ejection fraction (LVEF) was 61.10 ± 7.58%.

**Table 1:** Baseline characteristics of the study cohort.

Variables	Statistical results
Age (years)	69 (44–87)
Male sex	107 (63.3%)
Height (cm)	165.72 ± 7.45
Weight (kilograms)	67.68 ± 12.62
Body mass index	24.29 ± 3.59
Atrial fibrillation type	
Paroxysmal	45 (26.6%)
Persistent or permanent	124 (73.4%)
CHA2DS2-VASc score	4.31 ± 1.54
HAS-BLED score	2.74 ± 1.15
Comorbidity	
Hypertension	118 (69.8%)
Diabetes mellitus	34 (20.1%)
Coronary artery disease or vascular disease	123 (72.8%)
Congestive heart failure	31 (18.3%)
Previous stroke or TIA	96 (56.8%)
Echocardiography	
Left atrial diameter (mm)	44.09 ± 7.55
Left ventricular end diastolic diameter (mm)	48.25 ± 5.01
Left ventricular ejection fraction (%)	61.10 ± 7.58

### 3.1 Intra-procedural measurements

The maximum LAA ostium diameter, maximum LAA depth, and 1st sheath marker band length were measured in all patients using the delivery sheath, pigtail sheath, and steel ball as reference scales, respectively, which we called the delivery sheath group, pigtail sheath group and steel ball group. The maximum LAA ostium diameter and maximum LAA depth were also measured by TEE. The mean maximum LAA ostium diameter measured in steel ball group was 24.73 ± 3.39 mm, which was significantly higher than that in delivery sheath group (20.04 ± 3.24 mm, *p* < 0.001) and pigtail sheath group (22.48 ± 3.74 mm, *p* < 0.001), but was not significantly different from the results measured by TEE (24.39 ± 4.13 mm, *p* = 0.176); similarly, the mean maximum LAA depth measured in steel ball group was 27.64 ± 4.47 mm, which was higher than that in delivery sheath group (22.40 ± 4.02 mm, *p* < 0.001) and pigtail sheath group (22.48 ± 3.74 mm, *p* < 0.001), but was not different from the results measured by TEE (27.19 ± 5.04 mm, *p* = 0.113). The mean 1st sheath marker band length measured in steel ball group was 20.71 ± 0.61 mm, which was also significantly higher than that in delivery sheath group (16.78 ± 1.42 mm) and pigtail sheath group (18.83 ± 1.90 mm) (both *p* < 0.001); and the difference between 1st sheath marker band length measured in steel ball group and the true length (21 mm) was 0.29 ± 0.61 mm, which was significantly less than that in delivery sheath group (4.22 ± 1.42 mm) and pigtail sheath group (2.17 ± 1.90 mm) (both *p* < 0.001) (Tables 2, 3).

**Table 2:** Measurement results of the delivery sheath group, pigtail sheath group, and steel ball group.

Measurement results	Delivery sheath group	Pigtail sheath group	Steel ball group	*p* value
Mean maximum LAA ostium diameter (mm)	20.04 ± 3.24	22.48 ± 3.74	24.73 ± 3.39	<0.001
Mean maximum LAA depth (mm)	22.40 ± 4.02	25.08 ± 4.46	27.64 ± 4.47	<0.001
Mean 1st sheath marker band length (mm)	16.78 ± 1.42	18.83 ± 1.90	20.71 ± 0.61	<0.001
21 minus 1st sheath marker band length (mm)	4.22 ± 1.42	2.17 ± 1.90	0.29 ± 0.61	<0.001

**Table 3:** Comparison of results measured by steel ball and TEE.

Measurement results	Measured by Steel ball	Measured by TEE	*p* value
Mean maximum LAA ostium diameter (mm)	24.73 ± 3.39	24.39 ± 4.13	0.176
Mean maximum LAA depth (mm)	27.64 ± 4.47	27.19 ± 5.04	0.113

### 3.2 Morphologies of LAA and Watchman device implantation

The most common morphology of LAA in the study was cauliflower (74.6%), followed by chicken wing (18.9%), 3.6% was Cactus and 2.9% was Windsock. All Watchman devices were selected based on the measurement of steel ball group. Generally, the size of Watchman device was selected to be 3–5 mm larger than the measured maximum LAA ostium diameter in steel ball group, according to our center's experience. In the study, 75.2% of Watchman devices were implanted successfully with one release, and 88.2% of cases had no more than two releases. The median size of implanted Watchman device was 30 (21–33) mm, with an implantation success rate of 98.8% (169/171). The mean procedural time was 56.56 ± 25.59 min, while the mean x-ray exposure time was 7.53 ± 3.99 min (Table 4). The mean difference between size of implanted Watchman device and maximum LAA ostium diameter measured in steel ball group was 4.24 ± 2.46 mm, which was significantly less than that in delivery sheath group (8.97 ± 2.91 mm, *p* < 0.001) and pigtail sheath group (6.53 ± 3.31 mm, *p* < 0.001) (Table 5). No residual PDL was detected in 91.1% (154/169) of patients with immediate post-procedural TEE scan, 13 patients (7.7%) had a PDL ≤ 3.0 mm, 1 (0.6%) had a PDL between 3.1 and 5.0 mm, and only 1 (0.6%) had a PDL > 5 mm (Table 6).

**Table 4:** Characteristics and parameters during LAAC procedure.

Intra-procedural data	Statistical results
Morphologies of LAA	
Cauliflower	126 (74.6%)
Chicken wing	32 (18.9%)
Cactus	6 (3.6%)
Windsock	5 (2.9%)
Time of LAAC procedure (min)	56.56 ± 25.59
Time of x-ray exposure (min)	7.53 ± 3.99
Number of releases of Watchman device	
1	127 (75.2%）
2	22 (13.0%)
≥3	20 (11.8%)
Median size of implanted Watchman device (mm)	30 (21–33)
Post-procedural anticoagulation strategy	
Warfarin	12 (7.1%)
Rivaroxaban	86 (50.9%)
Dabigatran	69 (40.8%)
Others	2 (1.2%)

**Table 5:** Differences between actual size of Watchman device implanted and the measured maximum LAA ostium diameter in the delivery sheath group, pigtail sheath group, and steel ball group.

Measurement results	Delivery sheath group	Pigtail sheath group	Steel ball group	*p* value
Differences between size of Watchman device implanted and maximum LAA ostium diameter (mm)	8.97 ± 2.91	6.53 ± 3.31	4.24 ± 2.46	<0.001

**Table 6:** PDL detected by TEE immediately and 45 days after procedure.

PDL detected by TEE	Immediately after procedure	45 days after procedure
None or ≤3 mm	167 (98.8%)	164 (97.0%)
>3 and ≤5 mm	1 (0.6%)	4 (2.4%)
>5 mm	1 (0.6%)	1 (0.6%)

### 3.3 Complications and follow-up

2 patients (1.2%) occurred pericardial tamponade post-procedure, whose measured maximum LAA ostium diameter was 28.46 mm and 28.00 mm by “steel ball method”, respectively, and a 33 mm Watchman device was implanted in both cases. Both patients were cured after pericardiocentesis. After 45 days of follow-up, TEE was performed again to evaluate the Watchman device in all patients. 97.0% of patients had either no or slight (≤3 mm) PDL, 4 patients (2.4%) had a PDL between 3.1 and 5.0 mm, and only 1 (0.6%) had a PDL > 5 mm (Table 6). DRT on surface of Watchman device was found in 1 patient (0.6%) which did not cause stroke, while the rest of patients did not have DRT or other complications related to LAAC. Ischemic stroke occurred in 2 patients (1.2%) during follow-up, but no left atrial thrombus or DRT was found in both 2 patients on TEE of 45 days, which we considered might be non-cardiogenic stroke.

## 4 Discussion

We investigated the efficacy and safety of a novel method using body surface steel ball as a reference scale to measure the LAA, which may have potential advantage compared to using the traditional “sheath method” and TEE during procedure of LAAC with Watchman device in AF patients. As we know, the “sheath method” was widely used in LAAC procedure in China, but some operators found that using the delivery sheath or pigtail sheath as a reference scale had some disadvantages. The measurement results could be affected by the systolic/diastolic deformation of different sheaths *in vivo* and the different display diameters of sheaths under different x-ray fluoroscopic angles. Some clinical studies had reported favorable results that use of pre-procedural left atrial CTA, intra-procedural TEE under general anesthesia, or intra-procedural ICE to measure the size of LAA, however, each of these methods had its limitations in terms of measurement accuracy, patient physiological state tolerance, ease of surgical operation, popularity of the technology, and economic perspective. The steel ball has the advantages of being non-deformable and impermeable to x-rays, and the use of body surface steel ball does not add to the patient's discomfort, the difficulty of intra-procedural maneuvers, or the additional economic burden. The location of the steel ball placement was chosen at the pulmonary valve auscultation zone because it has a clear skeletal markings (the second intercostal space at the left edge of the sternum), and it does not interfere with intraprocedural electrocardiographic monitoring and positioning of ECG leads and does not obscure the operative area under x-ray fluoroscopy during procedure. And the steel ball placed in this position has the same depth as LAA in the x-ray projection at RAO30° + CAU20°, so the measurement of the relevant size of the LAA will not be affected by a different magnification ratio.

Since the 1st sheath marker band on delivery sheath of WATCHMAN device is length-fixed (21 mm) and impervious to x-ray, we used this as a reference to test the accuracy of the “steel ball method” and “sheath method”. In our study, the difference between 1st sheath marker band length measured in steel ball group and the true length (21 mm) was 0.29 ± 0.61 mm, which was significantly less than that in delivery sheath group (4.22 ± 1.42 mm) and pigtail sheath group (2.17 ± 1.90 mm) (both *p* < 0.001). It proved that the measurement results of “steel ball method” are more accurate than those of “sheath method”. The mean maximum LAA ostium diameter measured by steel ball was not significantly different from the results measured by TEE (24.73 ± 3.39 vs. 24.39 ± 4.13 mm, *p* = 0.176), which suggested the selected size of Watchman device would not be different by using the “steel ball method” or TEE. Considering some of the advantages of the “steel ball method”, we expect that it may potential be an alternative to the TEE for simplified and efficient LAAC procedures in the future, which may need more advanced researches.

Bode WD et al. reported the overall success rate of device implantation in LAAC was 93.9% with a meta-analysis study in 2015, which enrolled 1759 patients of 16 clinical researches who underwent LAAC (11). And in recent years, some clinical studies with large numbers of enrolled patients have also reported that the success rate of device implantation in LAAC ranges from 94.3%–98.5% (12–14). In our study, Watchman devices were all selected based on the measurement of steel ball group. According to our center's experience, the size of Watchman device would be selected to be 3–5 mm larger than the measured maximum LAA ostium diameter in steel ball group. Finally, the device implantation success rate of the study is 98.8%, with no serious intra-procedure complications in any of the patients. 75.2% of Watchman devices were implanted successfully with one release, and 88.2% of cases had no more than two releases. Only 2 patients (1.2%) experienced pericardial tamponade after procedure, both were cured after pericardiocentesis. 98.8% and 97.0% of patients had either no or slight (≤3 mm) PDL with immediate and 45 days post-procedural TEE scans, respectively. DRT was detected in just 1 patient during a routine 45-day post-procedural TEE scan, which did not cause stroke, fortunately.

The above findings suggest that using body surface steel ball as a reference scale to measure the left atrial appendage for optimal Watchman device sizing is accurate, effective, and safe. In our opinion, the “steel ball method” can be safely and easily integrated into current LAAC procedural workflows, and has a very short learning curve for operators. It does not require a lot of technological modifications, the extra needed during LAAC procedure is only a steel ball. The mean maximum LAA ostium diameter measured with “sheath method” was significantly smaller than that with “steel ball method” (*p* < 0.001), while the mean difference between size of implanted Watchman device and maximum LAA ostium diameter measured with “sheath method” was significantly larger than that with “steel ball method” (*p* < 0.001). We hypothesize that if Watchman devices are selected based on the measurement with “sheath method”, it is very likely that the size of Watchman device implanted may be too small, which can result in an increase in the incidence of post-procedural PDL, larger PDL, or even failure of operation.

### 4.1 Limitations

This was a retrospective single-center study. To standardize the measurement criteria of different measurement methods and to facilitate statistical comparisons, only patients implanted with the WATCHMAN device and used 12Fr delivery sheath (4 mm in diameter) and 5Fr pigtail sheath (1.67 mm in diameter) during procedure were enrolled in this study, which might lead to selection bias. Whether this result is applicable to other types of sheaths or devices for LAAC will have to be researched in further clinical studies. The steel ball placed at the pulmonary valve auscultation zone has the same depth as LAA in the x-ray projection at RAO30° + CAU20°, so the measurement of the relevant size of the LAA will not be affected by a different magnification ratio. However, in patients with particularly thick chest walls or when the angle of x-ray projection varies greatly, there may be some error in the “steel ball method”, although these cases are rare. In the longer post-procedural follow-up, the time of TEE examination varied considerably among patients, so we only counted the results of TEE 45 days after procedue.

## 5 Conclusions

The novel method using body surface steel ball as a reference scale to measure the left atrial appendage and guide the selection of Watchman device is accurate, effective, and safe in procedure of LAAC. It does not add to the patient's discomfort, the difficulty of intra-procedural maneuvers, or the economic burden. In our opinion, the size of Watchman device may be too small if selection is based on the measurement results using the delivery sheath or pigtail sheath as a reference scale, which can lead to unsatisfactory outcome of the procedure.

## Data Availability

The original contributions presented in the study are included in the article/Supplementary Material, further inquiries can be directed to the corresponding author.
